# SARS-CoV-2 and Coronavirus Disease Mitigation: Treatment Options, Vaccinations and Variants

**DOI:** 10.3390/pathogens11020275

**Published:** 2022-02-20

**Authors:** Almu’atasim Khamees, Jamal Bani-Issa, Mazhar Salim Al Zoubi, Taqwa Qasem, Manal Issam AbuAlArjah, Sura Ahmad Alawadin, Khayry Al-Shami, Farah E. Hussein, Emad Hussein, Ibrahim H. Bashayreh, Murtaza M. Tambuwala, Mohannad Al-Saghir, Christopher T. Cornelison

**Affiliations:** 1 Department of Clinical Sciences, Faculty of Medicine, Yarmouk University, Irbid 211-63, Jordan; almotasem.kh@gmail.com (A.K.); jamal.bani.issa.md@gmail.com (J.B.-I.); khyree20000@gmail.com (K.A.-S.); 2021999358@ses.yu.edu.jo (F.E.H.); 2 Department of Basic Medical Sciences, Faculty of Medicine, Yarmouk University, Irbid 211-63, Jordan; mszoubi@yu.edu.jo (M.S.A.Z.); takwaqasem95@gmail.com (T.Q.); manalessam12@yahoo.com (M.I.A.); 3 Faculty of Pharmacy, Yarmouk University, Irbid 211-63, Jordan; suraahmad95@gmail.com; 4 Department of Food Science and Human Nutrition, A’Sharqiyah University, P.O. Box 42, Ibra 400, Oman; emad.hussein@asu.edu.om; 5 Department of Biological Sciences, Faculty of Sciences, Yarmouk University, Irbid 211-63, Jordan; 6 Nursing Department, Fatima College of Health Sciences, Al-Ain Campus, P.O. Box 24162, Abu-Dhabi 31201, United Arab Emirates; ibrahim.bashayreh@fchs.ac.ae; 7 School of Pharmacy and Pharmaceutical Science, Ulster University, Coleraine BT52 1SA, UK; m.tambuwala@ulster.ac.uk; 8 Department of Biological Sciences, Ohio University, Zanesville, OH 43701, USA; al-saghi@ohio.edu; 9 Department of Molecular and Cellular Biology, Kennesaw State University, Kennesaw, GA 30144, USA

**Keywords:** 2019-nCoV, COVID-19, vaccines, variants, pandemic

## Abstract

COVID-19 is caused by a novel coronavirus (2019-nCoV), which was declared as a pandemic after it emerged in China 2019. A vast international effort has been conducted to prevent and treat COVID-19 due to its high transmissibility and severe morbidity and mortality rates, particularly in individuals with chronic co-morbidities. In addition, polymorphic variants increased the need for proper vaccination to overcome the infectivity of new variants that are emerging across the globe. Many treatment options have been proposed and more than 25 vaccines are in various stages of development; however, the infection peaks are oscillating periodically, which raises a significant question about the effectiveness of the prevention measures and the persistence of this pandemic disease. In this review, we are exploring the most recent knowledge and advances in the treatment and vaccination options as well as the new emerging variants of 2019-nCoV and the possible mitigation of one of the most aggressive pandemics in the last centuries.

## 1. Introduction

Novel coronavirus (2019-nCoV) or the severe acute respiratory syndrome coronavirus 2 (SARS-CoV-2) causes a disease known as coronavirus disease 2019 (COVID-19). SARS-CoV-2 belongs to the *Coronaviridae* family, a group of associated viruses with a single-stranded RNA that may cause respiratory infection with mild to lethal symptoms [1,2]. It was first recognized in Wuhan City, Central China, in December 2019, then spread quickly over the world, resulting in millions of deaths with an 11% mortality rate [3,4,5]. Coronavirus is an enveloped and non-segmented virus that has small crown-shaped spikes projecting from its surface with a size between (80–160 NM) [6]. The World Health Organization (WHO) considered SARS-CoV-2 as one of the most highly pathogenic β-coronaviruses infecting humans. Therefore, early diagnosis of COVID-19 is the most crucial step in treating the infection. Molecular methods were the most generally used tool in diagnosis, such as PCR [7]. After diagnosis, suspected and confirmed cases should be immediately treated in specially designated and supported hospitals with professional isolation and safe conditions [5]. Control of acute hypoxic respiratory failure in COVID-19 patients is the first step in treatment. Many trials were conducted to find an effective and safe therapeutic candidate. For instance: dexamethasone therapy was effective in reducing the mortality rate in patients requiring oxygen supplements [8,9]. This review summarizes the most effective drugs that have been used and evaluated for COVID-19 treatment during the pandemic and current knowledge about available vaccines and vaccine candidates.

## 2. The Pathogenic Mechanisms That Lead to Severe Lung Disease

Although more than two years have passed since the COVID-19 pandemic, the mechanism of the disease and its spread are still unclear [10]. In general, the mechanism of the disease can be divided into three stages:

The first stage, which can be referred to as an asymptomatic state:

The SARS-COV-2 virus is linked to the epithelial cells in the nasal cavity, where it begins to multiply. At this stage, the patient has no symptoms, and the presence of the virus can be confirmed by nasal swabs. This stage is characterized by the fact that even if the patient does not show symptoms, they are capable of transmitting the virus. The RT-PCR value can be used to predict viral load and subsequent infection [10].

The second stage:

The virus travels through the airways and an innate immune response occurs. At this stage, the presence of the virus can be confirmed through nasal or sputum samples, as well as by signs of the innate immune response and clinical symptoms. The level of CXCL10, which is an interferon-responsive gene, is used as a predictor of the clinical course and an indication of the noise-ratio of type II squamous cell responses to respiratory infections such as MERS-CoV and influenza [11,12].

The third stage:

Only 20% of the infected people reach this stage when they develop a pulmonary infiltration. It is estimated that the mortality rate is 2%, and varies according to the age of the infected person. At this stage, the virus reaches the gas exchange units inside the lungs, where it infects the second type of warm cells, where the virus is concentrated inside this type of cells and uses them as an amplification site, as these cells die when the virus is released. In this way, the lung will experience a significant loss of type II cells, which leads to the activation of a secondary healing pathway. Consequently, this leads to abnormal healing that results in scars and fibrosis inside the lungs. Older adults are most susceptible and most affected by this stage due to a weak immune response and a low rate of epithelium repair [10,12,13]. Moreover, Muratori et al. studied the effect of the presence of antinuclear antibodies in patients with moderate to severe COVID-19. They found that patients with antinuclear antibodies (positive-ANA) had a significantly worse COVID-19 prognosis than those who did not have these antibodies (negative-ANA) [13].

## 3. Treatments

### 3.1. Usual Care and Respiratory Support

The National Institutes of Health (NIH) provided guidelines for the supportive management of acute hypoxic respiratory failure caused by COVID-19 [14,15,16]. Approximately 75% of COVID-19 hospitalized patients required oxygen supply therapy. On the other hand, for patients who require invasive mechanical ventilation, a lung-protective ventilation approach with low tidal volumes around (4–8 mL/kg) and plateau pressure lower than 30 cm H_2_O is strongly recommended with an application of positive end-expiratory pressure (PEEP) in moderate to severe cases [15]. The relationship between COVID-19 and respiratory failure is still being resolved. Many patients had normal breathing parameters but were severely hypoxemic [17].

### 3.2. Pharmaceutical Drugs for COVID-19

Since the emergence of the 2019-nCoV pandemic, and due to its rapid spread, it was of utmost importance to resolve viral virulence and develop effective therapeutics. Primary treatment started in China by using intravenous and oral antibiotics as the CoV appeared during the influenza season [18,19]. To date, no reliable evidence of an effective drug against 2019-nCoV has emerged [20]. Many therapeutics were used to treat previous coronaviruses, including SARS and MERS, such as ribavirin, nitazoxanide, penciclovir, nafamostat, chloroquine, corticosteroid, as well as the most common antiviral agents like Remdesivir and favipiravir. Only two of these therapeutics (chloroquine and Remdesivir) were validated with high efficiency in treating SARS-CoV-2 infection, ex vivo [7,21].

#### 3.2.1. Chloroquine

**Figure 1:**
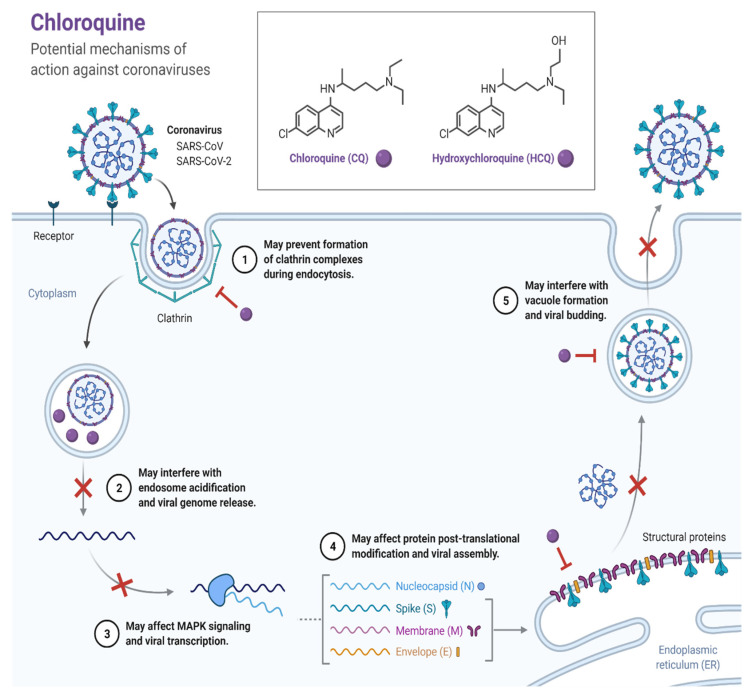
The mechanisms of action of chloroquine against coronaviruses. (Created with BioRender.com, accessed on 10 January 2022).

Several studies demonstrated the positive impact of the sulfate and phosphate salts of chloroquine, a broadly used antimalarial and autoimmune disease therapeutic in the treatment of patients with COVID-19 [22,23]. The antiviral activity of chloroquine was first demonstrated in vitro in the 1960s with several types of viruses [24,25,26]. It has been evaluated in many chronic viral diseases. For example, chloroquine was utilized in the treatment of HIV patients but was ultimately ineffective [27]. In clinical viral therapy, chloroquine was only effective against chronic hepatitis C [28]. Chloroquine demonstrated its inhibitory impacts against COVID-19 by enhancing the endosomal pH required for the fusion of viral cells. Additionally, it induced the glycosylation of cellular receptors of SARS-CoV-2 (Figure 1) [29]. Approximately 200 trials of chloroquine/hydroxychloroquine suppressed in vitro entry and endocytosis of the virus [30,31]. Wang et al. concluded that “chloroquine was highly effective for in vitro control of CoV-19 infection and it should be tested in human patients suffering from the novel coronavirus disease” [31].

Despite these aforementioned promising results, several large randomized clinical trials found no benefit to mortality or other clinical aspects in hospitalized COVID-19 patients treated with chloroquine/hydroxychloroquine [32,33,34,35,36]. One such study randomized 150 patients, of which two had severe disease, and 148 had mild to moderate disease, to either standard of care alone, or standard of care plus hydroxychloroquine, and did not find a significantly higher probability of negative conversion due to hydroxychloroquine and reported that adverse events were higher in hydroxychloroquine recipients than in non-recipients [33]. Furthermore, in June 2020, the United States Food And Drug Administration (FDA) revoked its emergency use authorization (EUA) for these agents in patients with severe COVID-19, noting that the known and potential benefits no longer outweighed the known and potential risks [37].

#### 3.2.2. Remdesivir

**Figure 2:**
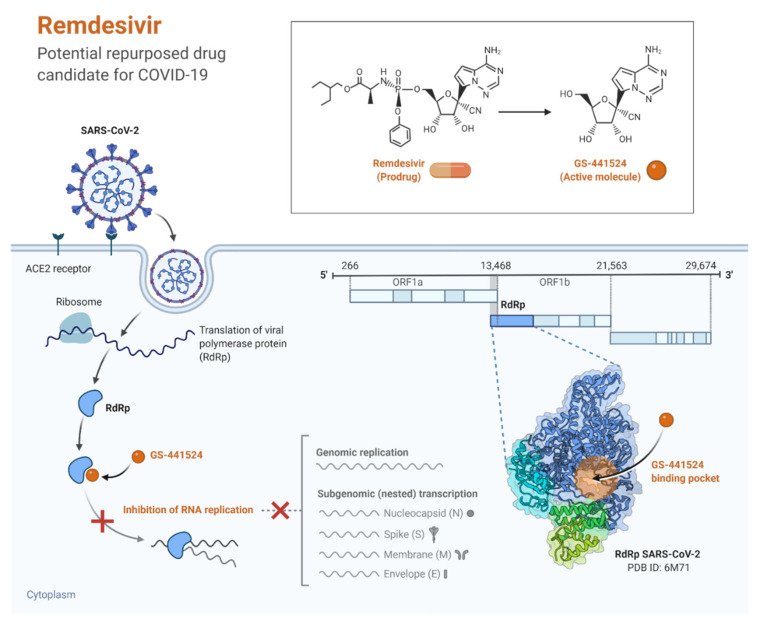
The mechanisms of action of Remdesivir against coronaviruses. (Created with BioRender.com, accessed on 10 January 2022).

Remdesivir is an antiviral prodrug and has activity against a broad spectrum of viruses, for instance, Nipah virus, Ebola virus, middle east respiratory syndrome (MERS)-CoV, and severe acute respiratory syndrome (SARS)-CoV [38,39]. Since the 2019-nCov had a positive sense RNA and depended on RNA-dependent RNA RdRP polymerases for replication, drugs like Remdesivir were considered as candidate therapeutics for Coivd-19 treatment [40,41]. The active metabolite form of Remdesivir (GS-443902) is an adenosine analog that accumulates in cells and works as a viral RdRP inhibitor, and therefore inhibits viral RNA replication (Figure 2) [39,42].

In vitro studies showed that Remdesivir had a potential efficacy against 2019-nCoV [31,43]. One such study demonstrated that Remdesivir inhibited the viral reproduction in E96-cells infected by 2019-nCov [43]. Pre-existence in vitro studies illustrated that Remdesivir inhibited viral reproduction in several types of cells infected by SARS-CoV and MERS-CoV [44,45]. Additionally, another study in the rhesus macaque model infected by MERS-CoV reported that Remdesivir reduced the viral loads in the lungs, trachea, bronchi, tonsils, and mediastinal lymph nodes [46]. Nevertheless, the results from the experiment in rhesus macaque infected by SARS-CoV-2 and treated by Remdesivir were novel and only gave a glimmer of hope. Even though the outcomes from in vivo and ex vivo experiments suggested that Remdesivir may be a suitable treatment for COVID-19, they did not reflect that Remdesivir reduced the mortality rate or improved recovery time in patients with COVID-19. In May 2020, the United States food and drug administration (FDA) issued emergency use authorization (EUA) for Remdesivir. Since that time, many studies have been initiated with COVID-19 patients to evaluate the safety and efficacy of Remdesivir [38].

Beigel and colleagues designed a placebo-controlled trial with 1062 patients (541 patients received Remdesivir while 521 received placebo) for up to 10 days. The median recovery time for patients who received Remdesivir was shorter in comparison to the placebo group (10 days versus 15 days, respectively). By day 15, the mortality rate in the Remdesivir group and placebo group was 6.7% and 11.9, respectively. Beigel and colleagues concluded that Remdesivir was superior to placebo in reducing recovery time in hospitalized patients with COIVD-19 [47].

The WHO solidarity trial is an open-label randomized trial conducted in 30 different countries and included 11,266 hospitalized patients with COIVD-19 to examine the efficacy and safety of four antiviral drugs that might be active in COVID-19 treatment. The WHO solidarity trial results showed that neither Remdesivir nor the other antiviral drugs included in the study had the potential impact on the clinical improvement of patients [48]. In a placebo-controlled trial of over ten hospitals in Hubei, China, 232 patients with 2019-nCoV infection had been recruited (158 received Remdesivir and 78 received placebo). The findings of this trial demonstrated that Remdesivir was not associated with a reduction in recovery time or mortality rate [49]. Additionally, in a randomized open-label trial with no placebo control, 397 patients were subdivided into two courses (200 patients for 5 days and 197 for 10 days) and illustrated that the difference between the two courses was not significant [42].

Spinner et al. designed an experiment with a standard care group as a control group where 584 patients were subdivided into three subgroups (193 patients for a 10-day course, 191 patients for a five-day course, and 200 patients in standard care group). On day 11, patients in the five-day course had better clinical improvement than the standard care group, but the difference between patients in the 10-day course and patients in the standard care group was not statistically significant [41]. Another meta-analysis showed a significant improvement in the recovery rate for patients admitted to the hospital with severe COVID 19 symptoms and treated by 10 days of Remdesivir. Those patients showed improvement in their symptoms on the seventh day with no significant difference in the patients’ health condition on the twenty-eighth day. This indicates that Remdesivir is more useful at the beginning treatment plan [47].

#### 3.2.3. Baricitinib (As Adjunct to Remdesivir)

Baricitinib, a reversible Janus-associated kinase (JAK) inhibitor, interrupts the signaling of multiple cytokines implicated in COVID-19 immunopathology. In addition to its immunomodulatory effects, it is thought to have potent antiviral effects through interference in viral entry [50]. In the United States, the FDA issued an emergency use authorization (EUA) for baricitinib to be used in combination with Remdesivir in patients with COVID-19 who require oxygen or ventilatory support while noting that there is limited information on its use in patients receiving systemic corticosteroids [51].In a randomized trial of 1033 hospitalized adults with COVID-19, the use of baricitinib in combination with Remdesivir reduced time to recovery (defined as a hospital discharge or continued hospitalization without the need for oxygen or medical care), when compared with placebo plus Remdesivir (7 versus 8 days, RR for recovery 1.16, 95% CI 1.01–1.32). Among the 216 patients who were on high-flow oxygen or non-invasive ventilation at baseline, the median recovery time in the group receiving baricitinib was 10 days versus 18 with placebo (RR 1.51, 95% CI 1.10–2.08). However, among the 223 patients who were also on glucocorticoids during the trial, no difference in recovery time was detected [52].

Moreover, an observational retrospective study included 43 hospitalized patients with severe COVID-19 managed with everyday baricitinib for six days duration. Baricitinab showed a significant statistical clinical improvement (*p*-value < 0.01) in addition to a 100% survival rate after one month and two months. The average recovery time was 12 days from the beginning of baricitinab. This drug was found to be safe with no side effects associated with baricitinib, and there was a statistically significant improvement at day 14 for all poor prognostic factors like Interleukin-6, serum ferritin, platelet, lymphocyte count, D-dimer, and C-reactive protein (*p*-value < 0.05) [53].

#### 3.2.4. Corticosteroids

Corticosteroids are a branch of steroid hormones that are produced in the adrenal cortex of vertebrates. They are classified into two major classes: glucocorticoids and mineralocorticoids. They play essential roles in many physiological processes. Several examples include their function in stress response, immune response, regulation of inflammation, carbohydrate metabolism, and protein catabolism. The most dominant naturally occurring steroid hormones are corticosterone, cortisone, aldosterone, and cortisol. The adrenal cortex produces the main corticosteroids (cortisol and aldosterone) [54].

In the SARS pandemic, corticosteroids had demonstrated no survival benefit and possible harm on patients. For this reason, the WHO discouraged the utilization of corticosteroids for COVID-19 patients [55,56,57]. However, in China, they observed that using corticosteroids reduced the mortality rate among COVID-19 patients, so the interest in utilizing the corticosteroids was renewed and recommendations began to support the use of corticosteroids, namely dexamethasone, in the treatment of SARS-CoV-2 patients [15,58,59,60,61,62,63]. This paradigm shift was largely due to data from randomized trials that overall supported the role of glucocorticoids. In a meta-analysis of seven trials that included 1703 critical COVID-19 patients, glucocorticoids reduced 28-day mortality compared with standard care or placebo (32 versus 40 percent, odds ratio [OR] 0.66, 95% CI 0.53–0.82) and were not associated with an increased risk of severe adverse events [9]. Another systematic review and network meta-analysis of randomized trials through mid-August 2020 found that glucocorticoids were the only intervention with at least moderate certainty in a mortality reduction (OR 0.87, 95% CI 0.77–0.98) or risk of mechanical ventilation (OR 0.74, 95% CI 0.58–0.92) compared with standard care [64]. Most of the efficacy data on glucocorticoids in these meta-analyses are based on a large, randomized, open-label trial in the United Kingdom in which oral or intravenous dexamethasone reduced 28-day mortality among hospitalized patients compared with usual care alone [65]. Other glucocorticoids were also investigated. Individual trials of hydrocortisone in critically ill patients failed to demonstrate benefit [66,67]. Meta-analysis of three trials evaluating hydrocortisone found a non-statistically significant trend towards reduced 28-day mortality compared with usual care or placebo (OR 0.69, 95% CI 0.43–1.12) [9]. Furthermore, trials that evaluated the use of methylprednisone found no clear benefit. A randomized trial from Brazil, including 393 patients with suspected or confirmed severe COVID-19 (77 percent of whom were on oxygen or ventilatory support), found no difference in 28-day mortality rates with methylprednisone compared with placebo (37 versus 38 percent) [68]. Inhaled corticosteroids were also investigated as potential agents that may be of benefit in the outpatient setting. In the non-placebo-controlled steroids in COVID-19 (STOIC) trial, 139 adult patients with mild early COVID-19 were treated with inhaled budesonide (800 mcg twice daily) for an average of seven days or assigned usual care. Fewer patients in the group treated with inhaled budesonide required urgent medical evaluation or hospitalization at 28 days (1.4 versus 14.4 percent), reflecting a possible role of inhaled corticosteroids in the outpatient setting [69]. However, in the same context, a recent study demonstrated that treatment of COVID-19 patients with corticosteroids did not affect hospital mortality rate [70]. In comparison with patients who did not receive corticosteroids, the positive impact associated with their utilization was a decline in ICU admission rate. Nevertheless, steroids can enhance compliance and hypoxemia in ARDS patients [71]. In this sense, treatment with steroids may help reduce pressure on the Intensive Care Units [70].

#### 3.2.5. Vitamin D

Vitamin D deficiency is an increasing global public health issue, and the effects of low vitamin D levels are being explored in a variety of clinical settings, including respiratory viral diseases. Several studies in both children and adults have found an inverse relationship between vitamin D levels and the severity and mortality of respiratory viral infections [72,73,74,75]. A study revealed that individuals with positive PCR for SARS-CoV-2 had a low 25-hydroxyvitamin D 25-OHD level in comparison with individuals with a negative PCR [76]. Additionally, the concentration of 25-OHD in serum was lower in severe cases and higher in moderate cases [77,78]. Since then, many studies have suggested that supplementation of vitamin D may play a potential role in Covid-19 treatment. Numerous trials have been started to examine the efficacy of vitamin D as supplementation in the treatment of Covid-19 patients. The pathway by which vitamin D influences the pathogenesis of COVID-19 is still unclear. However, many hypotheses that explained the influence of vitamin D in COVID-19 pathogenesis have been suggested [79]. The deficiency of glucose-6-phosphate dehydrogenase enhances viral replication. Furthermore, G6PD deficiency can increase the infectiousness of human Coronavirus 229E [80], due to the critical role of G6PD in cellular redox state. Nevertheless, the expression of the G6PD gene is enhanced by vitamin D, so vitamin D can also possibly affect COVID-19 pathogenesis indirectly [81].

One of the major factors affecting morbidity and mortality in COVID-19 is inflammatory-related disorders [82]. The pro-inflammatory responses in viral infection may be minimized by vitamin D due to several pathways, including (i) selective suppression of inflammatory cytokines, (ii) decreased influx of leukocytes into inflammatory regions [75], (iii) immune cells including neutrophils, monocytes/macrophages and mast cell interplay with vitamin D [83], (iv) vitamin D can also help to boost memory and regulatory T-cells [84], (v) Activity of T lymphocytes is influenced by vitamin D [85].

A randomized, placebo-controlled study found that vitamin D reduces recovery time in COVID-19 cases. Furthermore, there was a substantial reduction in inflammatory markers [86]. A quasi-experimental study reported that vitamin D supplementation reduced the severity of disease and improved the survival rate within elderly COVID-19 patients [87]. On another hand, a placebo-controlled trial failed to find any significant association between vitamin D supplementation and reduction in recovery time for COVID-19 [88], and a randomized trial from Brazil evaluating a single dose of vitamin D3 (200,000 international units) versus placebo in 240 moderately ill hospitalized COVID-19 patients (mean baseline 25[OH]D 20.9 ng/mL [52 nmol/L]) found no difference in length of hospital stay, and no significant differences in secondary outcomes such as in-hospital mortality (7.6 versus 5.1 percent, mean difference 2.5 percent, 95% CI −4.1 to 9.2 percent), admission to the ICU (16 versus 21.2 percent, mean difference −5.2 percent, 95% CI −15.1 to 4.7 percent), or need for mechanical ventilation (7.6 versus 14.4 percent, mean difference −6.8 percent, 95% CI −15.1 to 1.2 percent) [88]. Nevertheless, several larger placebo-controlled trials evaluating the role of vitamin D are still in progress [89,90].

There is a debate about the benefit of using vitamin D to treat cases of COVID-19, as there are many studies that suggest a benefit from vitamin D use, while other studies reported no association. Currently, there is no conclusive evidence of a relationship between vitamin D levels and decreased mortality in COVID-19 patients [91].

#### 3.2.6. Interleukin-6 (IL-6) Pathway Inhibitors (e.g., Tocilizumab)

Markedly elevated inflammatory markers (e.g., D-dimer, ferritin) and elevated levels of pro-inflammatory cytokines (including interleukin [IL]-6) are associated with critical and fatal COVID-19 cases. Accordingly, it was suggested that mortality may be due to virally driven hyper inflammation [92]. A retrospective multicenter study on 150 confirmed COVID-19 cases in Wuhan, China, found that levels of ferritin (*p* < 0.001) and IL-6 (*p* < 0.0001) were higher in non-survivors compared with survivors and described such elevations to be predictors of mortality [93]. Preliminary results from an open-label international randomized trial that included 803 patients with severe COVID-19 who were admitted to the ICU and required respiratory or cardiovascular support suggest a mortality benefit of interleukin-6 pathway inhibitors [94]. In another 2021 trial that included 389 hospitalized patients with COVID-19 who were not on ventilatory support, tocilizumab reduced progression to mechanical ventilation or death at 28 days but not overall 28-day mortality [95]. Despite optimistic emerging preliminary evidence, several studies failed to show clear clinical benefit or reduced mortality associated with the use of interleukin-6 pathway inhibitors [95,96,97,98,99]. Trials that suggested a benefit with tocilizumab reported somewhat higher overall mortality rates when viewed in comparison with other trials, which may be interpreted to be a sign reflecting more severely affected populations. The National Institutes of Health (NIH) COVID-19 Treatment Guidelines Panel currently recommends adding tocilizumab to dexamethasone in recently hospitalized patients on high-flow oxygen or greater support that have either been admitted to the ICU within the prior 24 h or have significantly increased levels of inflammatory markers [58]. Moreover, the Infectious Diseases Society of America (IDSA) currently suggests adding tocilizumab to the standard of care (i.e., glucocorticoids) for hospitalized adults who have progressive severe or critical COVID-19 and have elevated markers of systemic inflammation [100].

#### 3.2.7. Interleukin-1 Pathway Inhibitors (e.g., Anakinra)

Interleukin-1 (IL-1) is a pro-inflammatory cytokine that has been associated with severe COVID-19. Several observational studies suggested that using IL-1 inhibitors such as anakinra is associated with reduced mortality in COVID-19 patients [101,102,103,104]. On the other hand, a randomized trial of 116 hospitalized patients with mild to moderate COVID-19 found no evidence of clinical benefit of anakinra addition to usual care compared with usual care alone and no statistically significant reduction in the rates of mechanical ventilation or death at 14 days was detected (34 versus 35 percent, respectively) [105].

However, a meta-analysis with 757 patients treated with anakinra while 2773 patients treated with standard non-anakinra management found that the usage of anakinra for treatment of COVID-19 patients was associated with a significant reduction in demand of mechanical ventilation and mortality rate compared with standard non-anakinra management. Although the anakinra group had slightly more risk of thromboembolism (OR = 1.59) and elevated liver transaminases enzymes (OR = 1.35) than the other group, these risks were not statistically significant, and the difference may be due to the characteristics of the control group [106].

More data from larger randomized trials are required to better understand the role of IL-1 inhibitors in the clinical resolution of COVID-19, in addition to resolving dosing procedures and potential side effects.

#### 3.2.8. Interferons (e.g., Interferon Beta)

Interferons function by modulation of immune responses and have been described to have antiviral effects [107]. Interferon-beta (IFN-β), specifically, has been reported to inhibit SARS-CoV-2 replication in vitro [108]. Furthermore, defects in the production of type I interferons (which include IFN-β) and autoantibodies that neutralize type I interferons have been identified in patients with severe COVID-19 [109,110]. Interim results of the WHO Solidarity Trial showed no difference in 28-day mortality with subcutaneous or intravenous interferon beta compared with usual care (2703 patients in each group; RR 1.16, 95% CI 0.96–1.39) [48]. Smaller trials reported clinical improvement, faster discharge times, and a potential mortality benefit with interferon beta [104]. However, methodologic limitations in such studies invoke reservations [111]. 

In addition, the debate about the effectiveness of IFN as a treatment for COVID-19 patients was due to the differences in the COVID-19 severity determination, time of IFN administration, and how the researchers read the test results either direct IFN effect or cellular reactions to IFNs [112]. In a retrospective cohort study with 446 COVID-19 patients in Hubei, China, they found that early initiation of IFN on the first five days of their hospital admission had a shorter hospitalization duration (*p*-value was 0.001), early improvement of CT scan findings (*p*-value was 0.001), and short COVID-19 duration from the beginning of symptoms to the end of hospitalization (*p*-value was 0.025). On the other hand, the delay in INF administration slowed recovery and increased mortality [113]. IT was also found that the prolonged administration of IFN therapy can lead to regeneration impairment or damage for the lung epithelium, and second bacterial infection superimposed COVID-19 infection, which may be lethal [113]. Accordingly, Jack Major et al. recommended usage of IFN treatment or prophylaxis by early strong administration of IFN to stop viral replication, followed by INF down-regulation to enhance regeneration of lung epithelium. The duration and timing of IFN administration are crucial for optimal effect, but more clinical studies regarding the efficacy and safety of using IFN for COVID-19 treatment are needed [114].

#### 3.2.9. Ivermectin

Ivermectin, a drug first discovered in a golf course in Japan by microbiologist Satoshi Ōmura in 1970, is one of the best-known antiparasitic drugs for its efficacy in treating a broad spectrum of parasitic infections [115]. In vitro studies on ivermectin at doses that far exceed those achieved in vivo with safe doses displayed in vitro activity against SARS-CoV-2, and thus it has been proposed as a potential therapy in COVID-19 [116]. Kaur et al. used artificial intelligence to study the interaction between ivermectin and SARS-CoV-2 proteins. They found a positive interaction between this drug and the viral proteins, which means it may have beneficial outcomes for COVID-19 patients [117]. In a retrospective review that included 280 hospitalized COVID-19 patients, administration of ivermectin was associated with a lower mortality rate. However, such patients were also more likely to receive corticosteroids, and thus, randomized studies are required to ascertain such positive effects in the absence of potential confounders [118].

#### 3.2.10. Favipiravir

**Figure 3:**
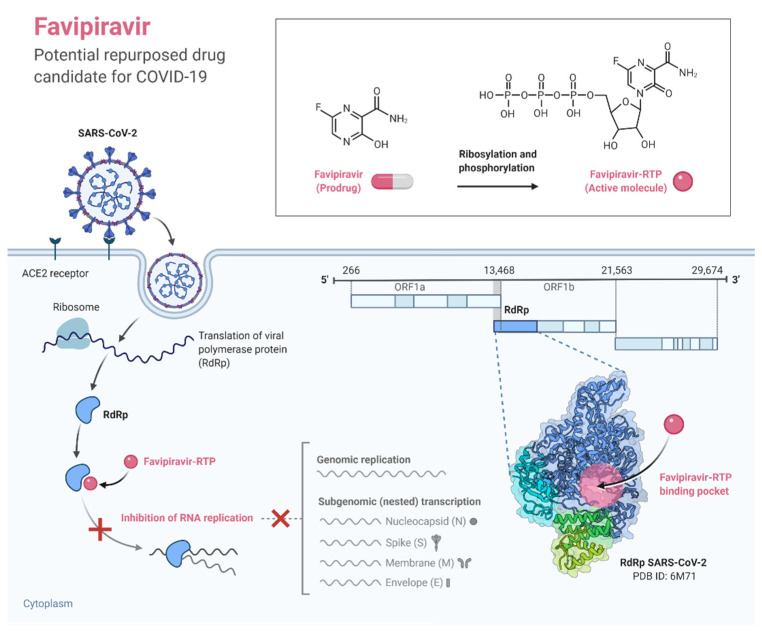
The mechanisms of action of Favipiravir against coronaviruses. (Created with BioRender.com, accessed on 10 January 2022).

Favipiravir is an RNA polymerase inhibitor licensed as an anti-influenza drug in Japan [119]. Favipiravir can effectively inhibit the RNA-dependent RNA polymerase of viruses such as influenza, Ebola, and norovirus [120]. Studies in Vero E6 cells have suggested that favipiravir can deactivate the SARS-CoV-2 virus (Figure 3) [31]. Early trials in which favipiravir was administered alongside other agents (e.g., immunomodulatory drugs) in Russia [121] and China [122] suggested some benefits. The role of favipiravir in COVID-19 treatment is being evaluated in clinical trials in the United States and elsewhere.

In a study with a total of 1489 Turkish patients, favipiravir alone was used in 537 patients while hydroxychloroquine alone was used in 545 patients, and a combination of both favipiravir and hydroxychloroquine was used for the rest of the patients. On the fourteenth day of follow-up, patients who were treated with hydroxychloroquine had improvement of COVID-19 symptoms by 1.8-fold greater than patients who were treated with favipiravir alone. There was a similarity in the rate of hospitalization among patients who were treated with hydroxychloroquine and favipiravir. However, among patients who had pneumonia at the time of COVID-19 diagnosis, those who were treated with hydroxychloroquine alone had a hospitalization rate of 6.6-fold less than patients who were treated with favipiravir alone and by 7.3-fold less than patients who were treated with the combination of drugs [123].

#### 3.2.11. Molnupiravir (Lagevrio)

Molnupiravir is an oral antiviral that has activity like Remdesivir and favipiravir. It can effectively inhibit the RNA-dependent RNA polymerase of viruses such as influenza and alphavirus infections [124,125,126]. Accordingly, the utility of molnupiravir in COVID-19 treatment is being evaluated by several researchers.

In a large systemic review, a daily dose of 1600 milligram molnupiravir for 5.5 days, which was safe with no significant side effects, can significantly decrease COVID-19 mortality rate at day 29 by 50% as well as decreased the hospitalization rate for mild COVID-19. There was no significant impact in moderate or severe COVID-19 infection [125]. Additionally, there was no effect of SARS-CoV-2 variants, symptoms onset time, or patients’ risk factors on the efficacy of molnupiravir [125]. Molnupiravir can also effectively decrease the nasopharyngeal SARS-CoV-2 virus and viral RNA isolation at day 3 and no virus was isolated at day 5 of administration [126,127].

As a result, the medicines regulator in the United Kingdom was the first country that approved molnupiravir for the management of adults with mild to moderate COVID-19 and has any risk factors for severe infection [128]. Subsequently, it has been approved in several countries such as the United States, Canada, China, Japan, Korea, and the Europe Union [129]. Moreover, molnupiravir was the first antiviral drug that can be taken orally, which is a significant advantage [129,130,131]. However, some experts have suggested that molnupiravir should be used for COVID-19 patients outside the hospital, while Remdesivir is the preferred choice for COVID-19 patients under hospital care [129].

#### 3.2.12. Lopinavir-Ritonavir

**Figure 4:**
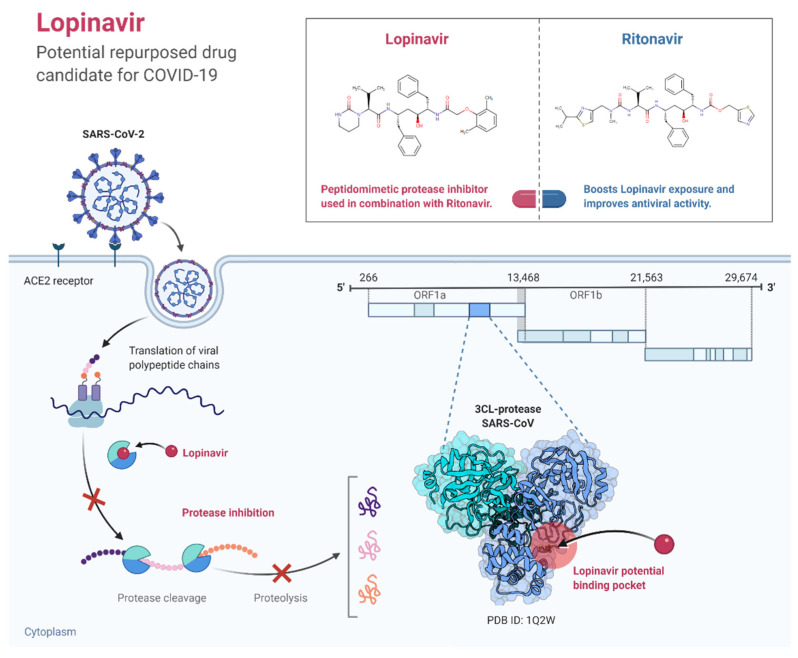
The mechanisms of action of lopinavir against coronaviruses. (Created with BioRender.com, accessed on 10 January 2022).

Co-formulated lopinavir-ritonavir is a boosted protease inhibitor (PI) that contains lopinavir and low-dose ritonavir. It is approved for use in combination with other antiretroviral drugs for the treatment of HIV in the United States and the European Union [130]. Several clinical trials demonstrated no efficacy of lopinavir-ritonavir in the treatment of COVID-19 patients [65,113,132,133]. One such study, which was an open-label randomized trial of hospitalized COVID-19 patients, found that lopinavir-ritonavir administration for up to 10 days did not reduce 28-day mortality or 28-day discharge rates, or the need for mechanical ventilation when compared with usual care (Figure 4) [134].

#### 3.2.13. Sofosbuvir Plus Daclatasvir

The role of concomitant use of Hepatitis C virus (HCV) antivirals sofosbuvir and daclatasvir has been explored in small-scale studies [131,132,135] One such study described that the addition of sofosbuvir and daclatasvir to standard care significantly reduced the duration of hospital stay compared with standard care alone [131]. Larger randomized trials are needed to fully understand the potential role of such agents in the management of COVID-19.

#### 3.2.14. Azithromycin

The macrolide antibiotic azithromycin has been proposed as a potential agent in the treatment of COVID-19 based on its immunomodulatory actions [133]. Several studies that evaluated the use of azithromycin alone or with hydroxychloroquine found no clinical benefit of its use in COVID-19 [35,133,136,137,138,139,140].

#### 3.2.15. Colchicine

The role of colchicine in the treatment of hospitalized COVID-19 patients was also investigated [141]. In March 2021, the RECOVERY trial closed recruitment to its randomized clinical trial that compared colchicine to usual care alone, which started in November 2020. The RECOVERY trial independent Data Monitoring Committee (DMC) recommended this action, saying that “The DMC saw no convincing evidence that further recruitment would provide conclusive proof of worthwhile mortality benefit either overall or in any pre-specified subgroup” [142].

On the contrary, a meta-analysis showed that colchicine had a clear positive outcome in reducing mortality in COVID-19 patients. This conclusion is based on a small number of papers and patients, which maybe not be sufficient to be a definitive outcome [143].

#### 3.2.16. Fluvoxamine

Limited data suggest that the selective serotonin reuptake inhibitor (SSRI) fluvoxamine may reduce progression to severe disease in early mild cases of COVID-19 [144,145]. Until now, fluvoxamine combined with physical therapy had a positive impact among COVID-19 patients in ICU [146]. However, large scale high-quality randomized trials are required to fully assess the potential of fluvoxamine in the battle against COVID-19 in outpatient and hospital settings.

## 4. Convalescent Plasma Therapy

While many studies conducted worldwide suggest that elderly individuals and patients with chronic disease, especially those with chronic lung disease, are at higher risk of developing SARS-CoV-2-related pneumonia (COVID-19) and respiratory failure [147], little information is available to elucidate whether immunocompromised patients or patients receiving immunosuppressive treatment are at higher risk. A systematic review article published in July 2020 evaluating 16 articles with 110 immunocompromised patients infected with SARS-CoV-2 suggested that both children and adults with immunosuppression seem to have a favorable disease when compared to the general population [148] However, an alarming report by Tepasse et al., described fatally persisting viremia without any sign of viral clearance in two patients who received rituximab two weeks before infection with SARS-CoV-2, suggesting that due to its effect that leads to complete B-cell depletion, rituximab may be associated with severe COVID-19 outcomes [149]. Interestingly, a case report by Clark et al. demonstrated rapid clinical improvement followed by viral clearance after administration of hyperimmune plasma in a hospitalized COVID-19 patient who had recently been treated with rituximab [150]. 

In the general population, several observational studies suggested great clinical benefit with early administration of high-titer convalescent plasma [151,152,153]. In the outpatient setting, a randomized, double-blind, placebo-controlled trial with high IgG titer convalescent plasma was given within 72 h after the onset of mild COVID-19 symptoms to certain high-risk adult outpatients (age ≥75 years or ≥65 years with one or more specific comorbidities [hypertension, chronic obstructive lung disease, diabetes mellitus on pharmacotherapy, cardiovascular disease, chronic renal failure, obesity]), found that early administration of high-titer convalescent plasma reduced the progression to severe disease [154]. However, randomized trials in hospitalized COVID-19 patients failed to demonstrate a clear clinical benefit of convalescent plasma [155,156,157,158]. One such placebo-controlled trial from Argentina included 333 cases with severe COVID-19 and found no statistically significant differences in clinical status at 30 days (adjusted odds ratio 0.92, 95% CI 0.59–1.42) or in 30-day mortality (10.96 versus 11.43 percent, risk difference −0.46 percent, 95% CI −7.8–6.8) between convalescent plasma (with a median total antibody titer of 1:3200) and placebo [158]. On February 4, the United States Food and Drug Administration (FDA) issued a revision of its original emergency use authorization (EUA) for the use of COVID-19 convalescent plasma that limits the authorization to the use of high titer COVID-19 plasma for the treatment of hospitalized patients in the early disease course [159]. It may also be useful in the reduction of hospital stay duration, mortality, the severity of symptoms, and even the recurrence of the side effects. Moreover, there is no definitive or strong significance about using this method in treatment [160].

## 5. Monoclonal Antibody Therapy (e.g., Bamlanivimab-Etesevimab; Casirivimab-Imdevimab)

Like SARS-CoV and MERS-CoV, SARS-CoV-2 is an enveloped, single-stranded, and positive-sense RNA virus. SARS-CoV-2 is phylogenetically related to SARS-CoV, sharing approximately 79.6% genomic sequence identity [161]. Similar to other emerging pathogenic human coronaviruses, the genome of SARS-CoV-2 encodes the spike (S) protein, which plays an essential role in viral attachment, fusion, entry, and transmission. The spike protein comprises an N-terminal S1 subunit responsible for virus-receptor binding and a C-terminal S2 subunit responsible for virus-cell membrane fusion. S1 is further divided into an N-terminal domain (NTD) and a receptor-binding domain (RBD). SARS-CoV-2 binds Angiotensin-converting enzyme 2 (ACE2) [162,163,164]. Virus neutralizing antibodies (nAbs) induced by vaccines or prior infection play crucial roles in controlling viral infection. Currently developed SARS-CoV and MERS-CoV-specific nAbs include monoclonal antibodies [165]. They target S1-RBD, S1-NTD, or the S2 region, thereby blocking the binding of RBDs to their respective receptors and interfering with S2-mediated membrane fusion or entry into the host cell [164,166]. SARS-CoV2-specific nAbs that target the host ACE2 receptor-binding domain show promise therapeutically [167,168,169,170,171] and are under clinical evaluation [172,173,174,175].

In November 2020, the United States FDA issued an emergency use authorization (EUA) for the use of basiliximab-imdevimab, which are investigational recombinant human IgG1 monoclonal antibodies that target the receptor-binding domain of the spike protein of SARS-CoV-2 [176]. On 9 February 2021, the U.S. FDA issued a EUA for the use of bamlanivimab-etesevimab, which are investigational neutralizing IgG1 monoclonal antibodies that bind to distinct but overlapping epitopes within the receptor-binding domain of the spike protein of SARS-CoV-2 [177]. Both EUAs were limited to combinational use only (e.g., bamlanivimab-etesevimab as opposed to either agent alone) in the treatment of mild to moderate COVID-19 in adults and pediatric patients (12 years of age and older weighing at least 40 kg) with positive results of direct SARS-CoV-2 viral testing, and who are at considerable risk for progressing to severe COVID-19 and/or hospitalization. The National Institutes of Health (NIH) treatment guidelines panel currently recommends treatment with combination bamlanivimab-etesevimab or casirivimab-imdevimab in high-risk outpatients as defined by the FDA EUA criteria [58]. The Infectious Disease Society of America (IDSA) currently recommends treatment only with bamlanivimab-etesevimab for such patients due to more limited data on the efficacy of casirivimab-imdevimab [178].

In phase 2/3 of BLAZE-1, a randomized controlled trial that included 577 patients with mild to moderate illness, different doses of bamlanivimab monotherapy, and combination bamlanivimab-etesevimab therapy were compared with placebo. At one month, treatment with combination therapy resulted in a small, statistically significant reduction in combined rates of emergency department visits or hospitalization (IRR 4.9%, 95% CI −8.9 to −0.8), and among participants who were ≥65 years of age or had a BMI ≥ 35 kg/m^2^, the risk of hospitalization with combination antibody therapy compared with placebo was significantly reduced (0 versus 13.5 percent) [179]. Furthermore, in an unpublished, preliminary report of results from a randomized controlled trial that included 4180 non-hospitalized adults with mild to moderate COVID-19, combination casirivimab-imdevimab, at two different doses, was compared with placebo. At 29 days, among patients with certain risk factors for severe COVID-19, there was a reduction in the combined outcome of hospitalizations and death among those treated with both doses of casirivimab-imdevimab compared with placebo (1200 mg total dose, 1 versus 3.2 percent; 2400 mg dose, 1.3 versus 4.6 percent) [180,181]. On the other hand, a randomized study that assigned 314 hospitalized patients who had COVID-19 without end-organ failure to either bamlanivimab or placebo found that the percentage of patients with the primary safety outcome was similar in the bamlanivimab group and the placebo group (19% and 14%, respectively; odds ratio, 1.56; 95% CI, 0.78 to 3.10; *p* = 0.20) [182].

The potency and safety of monoclonal therapy for children and teenagers are not proven, with inadequate evidence for some advantages among adult COVID-19 patients. Accordingly, a panel of pediatric infectious specialists recommended not to use monoclonal antibody therapy in children and teenager COVID-19 patients. Hypersensitivity and infusion reactions are possible side effects of this therapy [183].

Overall, further data is required to fully assess the possible role for monoclonal antibodies, and specifically, bamlanivimab-etesevimab and casirivimab-imdevimab, in the treatment of COVID-19 in different populations at outpatient and hospital settings.

## 6. Vaccines against COVID-19

Vaccinations are considered one of the most impactful inventions in medicine. Despite the success of many vaccines, new pandemics started at the end of the 20th century and the beginning of the 21st century, such as Human immunodeficiency virus (HIV) and SARS-CoV-2, respectively [184]. During the SARS-CoV-2 pandemic, several vaccines have been developed, and they are under continuous evaluation (Table 1). The WHO started the first vaccination program in December 2020, and at the time of this manuscript preparation, more than 983.1 million vaccine doses have been given worldwide [185]. Various generic platforms were used to develop the vaccines, such as using viral protein, Adenovirus-vectored vaccines, using an inactivated virus, mRNA, or RNA-based vaccine [184].

### 6.1. Adenovirus-Vectored Based Vaccines

The first trial was published in June 2020 [197]. The trial was a dose-escalation study of recombinant adenovirus type5 vectored S. Although around 75–83% of participants recorded mild or moderate symptoms, the vaccine was determined admissible [198].

The Oxford University/AstraZeneca’s adenovirus-vectored (AZD1222) vaccine was approved by the UK-Medicines and Healthcare Products Regulatory Agency [UK-MHPRA] in December 2020 and is considered as one of the most cost-effective vaccines, costing approximately 1.47−2.94£ per dose. AZD1222 is dependent on the chimpanzee adenovirus-vectored platform (ChAdOx1/AZD1222) encoding the spike glycoprotein of SARS-CoV-2. The application of the AZD1222 vaccine trial was done using two doses (5.0 × 10^10^ and 2.2 × 10^10^) of viral particles [199]. It was noticed that lowering the dose of viral particles had better clinical efficiency at around 90% in comparison with a 70% in the larger trial [200]. A third trial of the AZD1222 vaccine on 30,000 adults (20,000 vaccine recipients and 10,000 controls) started in August 2020 in many locations around the world. Due to the particular vulnerability of older ages against SARS-CoV-2, partly due to immunosenescence, AstraZeneca has been guiding its vaccine study and trial to clarify treatment efficacy and to measure neutralizing antibody levels (NAB) in seniors. To sum up, the side-effects were minor; AZD1222 triggered the induction of humoral responses by inducing anti-spike glycoprotein IgG nAbs, IFNγ, and T-cell responses, in most vaccines’ recipients after the first dose [201,202].

Among the Jordanian population who received this vaccine, only two cases of blood clot formation were reported to this date, and most vaccine recipients suffered from mild side effects (MOH report).

### 6.2. mRNA-Based Vaccines

Two RNA-based vaccines were approved for use by the United States FDA emergency use authorization (EUA) in December 2020. BNT162b2 manufactured jointly by Pfizer (Kalamazoo, MI, USA) and BioNTech (Mainz, Germany) with 95% efficiency, and mRNA-1273 manufactured by Moderna Company, Cambridge, MA, USA with 94.5% efficiency [200]. BNT162b1 is a lipid nanoparticle-formulated, nucleoside-modified mRNA encoding the receptor-binding domain of S protein. Antibodies that can recognize the receptor-binding domain and neutralize SARS-CoV-2 [203]. Similarly, mRNA-1273 also coded for stabilized prefusion S [204].

In comparison with other conventional vaccines, the process of approved mRNA-based vaccines (Pfizer/BioNTech and Moderna) was unique. While conventional vaccines utilize dead forms of the virus, which are grown in cell culture, these vaccines eliminate such a time-consuming process. Instead, mRNA vaccines include a non-viral “blueprint” mRNA that encodes specific instructions for the synthesis of a non-virulent spike protein in human cells. Using a lipid nanoparticle vehicle carrier (LNP) enhances the mRNA uptake by the human cell [205,206]. During vaccination, the synthetic spike protein triggers the host immune response by presenting on the outer membrane of the cell to involve neutralizing antibodies, cytotoxic CD8 cells, CD4 helper cells, and memory B cells. In a later stage, when the infection with SARS-CoV-2 occurs, its natural spike protein quickly produces the host’s own protective innate immune response, which had been previously sensitized by vaccination. It is important to stress the fact that mRNA did not enter into the nucleus, and no changes in cellular DNA occurred. The mRNA is degraded to a small molecule after the translation of its instructions [205].

### 6.3. Whole-Virus Inactivated Vaccines

**Figure 5:**
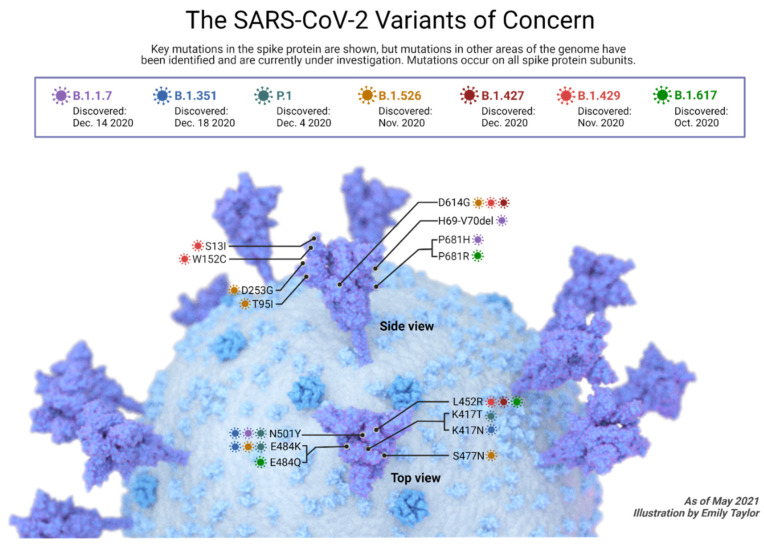
Common SARS-CoV-2 variants up to May 2021. (Created with BioRender.com, accessed on 10 January 2022).

These are conventional killed whole virus vaccines prepared in a way that had been utilized for many decades for different vaccines that are safe for breastfeeding mothers [207]. In such preparations, SARS-CoV-2 is not infectious as it is completely denatured after being grown in a cellular medium. When it is injected, the human body produces an immune response against the S protein that can neutralize the virus if the infection occurs [208]. Both the Chinese and Russian vaccines Sinopharm and Sinovac, respectively, are using the inactivated whole-virus method. They are simple to manufacture and suitable for people who have compromised immune systems. Among all these types of vaccines, it is difficult to guess which is more effective at persistent protection. Moreover, such viruses are highly polymorphic, which raises concern regarding the neutralizing ability of different vaccines on continuously emerging variants (Figure 5).

### 6.4. Subunit Vaccine

This type of vaccine does not pose a risk of causing disease inside the patient’s body because it does not contain live components of the virus. It contains the purified parts of the pathogen, such as proteins, or peptides, that are antigenic or necessary to form a protective immune response. This makes it safer, more stable, and suitable for people with weak immunity than other vaccines that contain the whole parts of the virus. However, the complexity of manufacturing this type of vaccine is one of the major disadvantages [209].

### 6.5. Other Types

There are other types of vaccines that are being tested by some trials, such as the multiplex plasmid DNA vaccines, the virus-like particle vaccines, and the conjugate vaccine. These available vaccines can limit the spread of the COVID-19 virus and increase immunity [209].

## 7. Discussion

The devastating effects on global public health, global economy, and psychological well-being of humans due to the COVID-19 pandemic are still being experienced worldwide. The latest figures from the World Health Organization show more than 386 million confirmed cases and over 5 million deaths globally. As researchers and scientists, we deal with mortality figures constantly, but over 5 million deaths reported to date create an unparallel sense of responsibility [185]. Humanity was not as well-prepared to meet this pandemic as many had presumed. Nevertheless, humans are once more demonstrating extraordinary efforts in the face of adversity. The continuous and creative efforts by researchers and clinicians worldwide to find reliable treatments for COVID-19 are nothing short of acts of heroism. The development of effective vaccines against COVID-19 in such a timeframe is indeed a great achievement for all humans. At the time of the manuscript preparation, more than 983.1 million vaccine doses have been given worldwide [185]. In this review, we aimed to recognize and shed light on the latest efforts and guide future researchers towards victory in the battle against COVID-19.

Studies on several drugs demonstrated promising results. Corticosteroids, namely dexamethasone, have been supported by several large, randomized trials, and current guidance recommends the use of dexamethasone in COVID-19. The definitive role of Remdesivir as a treatment for COVID-19 is still uncertain, with several studies demonstrating efficacy and the WHO solidarity trial demonstrating no clinical benefit. The co-administration of baricitinib with Remdesivir shows potential promise. Although, the use of baricitinib in patients being treated with corticosteroids requires further research. The examined literature demonstrated that vitamin D deficiency might be associated with severe COVID-19, and several studies demonstrated conflicting results on its use as a treatment in COVID-19 patients. The potential role of chloroquine/hydroxychloroquine as a treatment has been a matter of interest to clinicians around the world. The latest evidence from several large randomized clinical trials found no benefit to mortality or other clinical aspects in hospitalized COVID-19 patients treated with chloroquine/hydroxychloroquine. Furthermore, available data found no clinical benefit to the use of the macrolide antibiotic azithromycin alone or with hydroxychloroquine in COVID-19. Results concerning interleukin-6 inhibitors, particularly tocilizumab, spark interest in a potential role of tocilizumab in select cases of severe COVID-19 with elevated levels of inflammatory markers. The potential role of IL-1 inhibitors (e.g., anakinra), requires further research, however. Interim results of the WHO Solidarity trial demonstrate no efficacy of interferon-beta, although smaller limited trials reported benefit.

Emerging evidence on antibody-based therapies was indeed interesting. Emerging evidence supports a potential role for early administration of high-titer convalescent plasma in high-risk individuals in the outpatient setting, and case reports on its significant efficacy in patients previously treated with rituximab reveal new populations for further research. Preliminary evidence confers optimism towards novel SARS-CoV-2-specific monoclonal antibodies such as bamlanivimab-etesevimab and casirivimab-imdevimab, but the demonstration of their effect on different variants of SARS-CoV-2 requires further research efforts. Combination use of lopinavir-ritonavir also gathered attention as potential agents in COVID-19. However, several clinical trials demonstrated no efficacy of lopinavir-ritonavir in the treatment of COVID-19 patients. Another well-known drug, colchicine, was also evaluated in the literature. Emerging data suggest no benefit in hospitalized patients, but more trials are required to fully understand the possible role of colchicine in outpatient and hospital settings.

Other potential agents are also under evaluation. With great interest, we follow trials that are being conducted on the RNA polymerase inhibitor favipiravir, which has shown promise in early trials conducted in Russia and China. The well-known antiparasitic drug ivermectin, and the HCV antivirals sofosbuvir and daclatasvir may have possible roles in COVID-19 treatment, but high-quality data from large, randomized trials is needed, to ascertain such proposed roles.

### 7.1. The Role of Mathematical Models on COVID-19 Pandemic

To study and know the approximate estimation of the transmission, mathematical model-based analysis was used, which has had an important impact on the COVID-19 pandemic. It is considered a critical tool in the battle against the SARS-COV-2 virus by facilitating a better understanding of the mechanism of disease transmission, as well as improving the resolution of cost-effectiveness of different scenarios [210]. Mathematical models of past and current epidemics have been relied on to examine the mechanism and pathogenesis of disease spread, the severity of symptoms, suspected immune response, duration to reach the appropriate immune response, time to recovery, cost, complications, and how to contain the disease [211,212]. Of these patterns: deterministic models and stochastic models, whose groups are created utilizing population data in the region or independent data with details of movement between regions. Likewise, a mechanistic model is used to study and predict the efficiency of prophylactic actions such as lockdowns and social distancing. The existing and prospective risks of COVID-19 on lifestyle behaviors can be predicted using a community-level micromodel, especially for ending the lockdown and travel restrictions. Moreover, estimation, specification, prediction models, and other models have studied and analyzed the mechanism of virus transmission [212,213]. Despite the importance of mathematical models in limiting the spread of epidemics in general, and COVID-19 in particular, many challenges to the utility of mathematical models exist. Some examples of challenges include the difficulty of translating and applying the decision based on these models or mathematical motives to limit the spread of COVID-19 or any other pandemic, as well as efforts to verify the validity of mathematical models after each pandemic to examine the reliability of these models and increase the confidence of people, governments, and institutions in these models to increase utility [213,214].

### 7.2. Impact of COVID-19 Pandemic on Mental and Psychiatric Health and Vice Versa—A Special High-Risk Group

During the pandemic, the World Health Organization expressed its concern regarding the mental health of people and the impact of the pandemic on them. The COVID-19 precautionary measures such as isolation and quarantine led to an increase in feelings of anxiety, depression, and insomnia, and increased opportunities for alcohol and drug abuse, contributing to self-harm and thoughts of suicide [215]. The closures that occurred around the world led to an increase in cases of domestic violence due to the increase in the estimated time that the abuser interacts with their family, and this view is supported by a survey conducted by the Indian Society of Medicine, which indicated an increase in the incidence of mental illness by 20% [216]. In Kerala, India, withdrawal symptoms appeared and suicides by alcoholics increased after the closure of venues of alcohol sales.

As we are in a battle with the SARS-CoV-2 virus, some recommendations have obsessive-compulsive disorder triggers and some excessive behaviors such as procedures related to washing hands and cleaning surfaces to reduce the spread of the disease. In addition to some emotions such as intense anxiety, fear, and phobia of meeting others or fear of isolation or death as a result of this pandemic. Or even the fear of not getting food and essential items, as some people deliberately stock up on food [48]. COVID-19 patients who suffer from mental illnesses such as Alzheimer’s disease, dementia disorders, and other mental illnesses have an elevated mortality rate compared to patients who do not suffer from mental illnesses. This was shown in a meta-analysis study, where the higher mortality rate of COVID-19 patients among dementia patients was noticeable [1].

Although the diversity in the diagnosis of COVID-19 patients, age and chronic diseases were among the most important factors for predicting poor prognosis, interestingly, age and chronic diseases were also associated with dementia patients. Accordingly, dementia patients are more susceptible to diseases such as high blood pressure, diabetes, and pneumonia. If we assume the absence of the COVID-19 pandemic, the death rate due to pneumonia for dementia patients was twofold higher than people without dementia [217,218]. Another reason for the high death rate of COVID-19 in patients with dementia is the cytokine storm, where it has been shown that dementia patients have elevated proinflammatory cytokines, such as tumor necrosis factor-alpha, interleukin-1, and interleukin-6, which increases the death rate [219,220]. Another logical and simple reason for the high mortality of COVID-19 among dementia patients is that dementia patients may not be able to follow the advice and directions of health authorities, monitor and report symptoms of COVID-19, or even stay away from others to prevent the spread of infection [221]. 

Kubota T and Kuroda N conducted a systemic review on 2278 patients who had neurological diseases before the COVID-19 pandemic. They found that patients with dementia, Parkinson’s disease, epilepsy, multiple sclerosis, cerebrovascular diseases, spinal cord injury, and any preexisting neurological diseases were significantly associated with severe COVID-19 symptoms, worsening of their neurological status, and increased mortality rate [222]. These findings are in complete agreement with the study by Lanza G et al., who found that COVID-19 can worsen the psychiatric and neurological symptoms for all people and significantly more in chronic preexisting neuropsychiatric diseases [223]. Accordingly, authorities should give more attention to these high-risk groups.

## 8. Variants of Concern and Vaccines

Vaccines are largely considered to be the most effective intervention in the battle against COVID-19. The WHO started the first vaccination program in December 2020, and more than 983.1 million vaccine doses have been given worldwide, according to the WHO [185]. According to Our World In Data [224], 493.46 million doses were given in Asia, while 266.68 million doses were given in North America and 210.11 million doses in Europe. In China alone, 235.98 million vaccine doses were given, being the highest number among countries, followed by the United States with 232.41 million doses. Relative to population, Gibraltar has given 204.07 vaccine doses per one hundred citizens, achieving 95.06% of fully vaccinated individuals among the population, followed by Israel with 58.63%. In the middle east, the United Arab Emirates achieved 38.79%. The United States reached 28.93%, while the United Kingdom achieved 49.85%. The latest figures show that South Africa only achieved 0.49% of fully vaccinated individuals among the population.

Like other viruses, SARS-CoV-2 evolves. Certain variants have raised global concern because of their rapid emergence within populations. The emergence of such variants raised concern regarding the neutralizing ability of currently used vaccines against such variants (Figure 5).

### 8.1. B.1.1.7 Lineage (Alpha or GR/501Y.V1)

This variant was first identified in the United Kingdom in late 2020 and was temporally associated with an increase in regional infections [225]. Several epidemiologic analyses comparing growth rates of the B.1.1.7 variant with those of other strains in the United Kingdom suggest that the B.1.1.7 variant has a transmission advantage over wild-type strains [225,226,227]. A report from Public Health England found a secondary infection rate of 12.9% among 37,585 contacts of individuals with the B.1.1.7 variant infection compared with 9.7% among 24,239 contacts of individuals with wild-type infection and estimated that the increase in transmissibility associated with the B.1.1.7 variant was 25–40% [228]. In a study of nearly 55,000 individuals with B.1.1.7 infection, the risk of mortality was greater when compared with matched controls with wild-type infection (hazard ratio 1.64) [229]. However, the absolute mortality rates were low in both groups (approximately 0.4 versus 0.25 percent). In a smaller study of 496 hospitalized patients, there was no association between B.1.1.7 infection and severe disease and death, with and without adjustment for comorbidities and other potential confounders [230]. Plasma from trial participants vaccinated with BNT162b2 (Pfizer-BioNTech COVID-19 vaccine) appears to maintain neutralizing activity against B.1.1.7 [231]. Based on preliminary data, plasma from recipients of mRNA-1273 (Moderna COVID-19 vaccine) appears to maintain neutralizing activity against B.1.1.7 [232,233]. In an analysis of one of the randomized trials on ChAdOx1 nCoV-19/AZD1222 (University of Oxford, AstraZeneca, and the Serum Institute of India), efficacy against symptomatic COVID-19 causes by variant B.1.1.7 was not statistically different, when compared with other variants (70 vs. 82 percent). However, it induced lower neutralizing activity against the B.1.1.7 variant [234]. Furthermore, in a study that evaluated pseudovirus resistance to serum obtained from individuals that received two doses of inactivated-virus vaccines (BBIBP-CorV (Sinopharm) and CoronaVac (Sinovac)), B.1.1.7 showed little resistance to the neutralizing activity of vaccinee serum [235].

### 8.2. B.1.351 Lineage (Beta or GH501Y.V20)

This variant was identified in South Africa in late 2020 and spread rapidly to become the dominant variant in several South African provinces within weeks, suggesting possible increased transmissibility of this strain [236]. In a study that introduced the B.1.351 lineage spike protein into a viral construct, attenuation of neutralizing activity of convalescent plasma occurred, with 48 percent of plasma samples losing neutralizing activity [237]. Plasma from trial participants vaccinated with BNT162b2 (Pfizer-BioNTech COVID-19 vaccine) appears to maintain neutralizing activity against B.1.351, but with lower neutralizing titers (in one study, approximately two-thirds lower) than those for other circulating variants [238,239,240]. Plasma from recipients of mRNA-1273 (Moderna COVID-19 vaccine) appears to maintain neutralizing activity against B.1.351, but at up to six- to nine-fold lower titers than with wild-type virus [232,233]. In preliminary results of phase I/II trial in South Africa, ChAdOx1 nCoV-19/AZD1222 (University of Oxford, AstraZeneca, and the Serum Institute of India) did not reduce the rate of mild to moderate COVID-19 over a timeframe when B.1.351 was the dominant circulating variant, but the trial was small, and the number of cases was low, and the estimate of vaccine efficacy had wide confidence intervals (21.9 percent, 95% CI −49.9 to 59.8) [241]. Furthermore, in phase, I/II placebo-controlled randomized trials of healthy individuals 18 to 80 years old who received two doses of BBIBP-CorV (Sinopharm), all recipients of two vaccine doses developed neutralizing and binding antibodies against SARS-CoV-2 but demonstrated the same trend observed with other vaccines, where neutralizing activity against the B.1.351 was reduced [235]. Overall, vaccines remain effective against the B.1.351 lineage.

### 8.3. B.1.1.28 Lineage // [Lineage P.1 (B.1.1.28.1) (Gamma); Lineage P.2 (B.1.1.28.2) Zeta; P.7]

The expert estimations revealed that P.2 lineage (B.1.1.28.2 or Zeta) started in February 2020 and the first detection was in November in Rio de Janeiro. However, P.2 spread rapidly to arrive at its peak at the end of 2020. While P.1 (B.1.1.28.1 or gamma) emerged in August 2020 and the first detection was in December 2020 in Amazonas state. There was also evidence about P.7 that originated in south Brazil then spread rapidly to northern Brazil. These three lineages of B.1.1.28 (especially P.1 and P.2) co-dominated and made the second wave in Brazil [242].

In general, B.1.1.28 lineage contains 10 spikes mutations and shares the same three mutations in the receptor-binding domain (RBD) that found in the beta variant also [243]. A previous COVID-19 infection cannot be adequate to protect the population from this variant [244]. Although a relative resistance of SARS-CoV-2 variants B.1.1.7 and B.1.351 was reported to monoclonal antibodies, they are not similar to the B.1.1.28 lineage, which was resistant to monoclonal antibodies, convalescent plasma therapy, and vaccine sera [243]. Plasma from an 83-year-old man who was vaccinated with BNT162b2 (Pfizer-BioNTech COVID-19 vaccine) and diagnosed with B.1.1.28.1 (P1 lineage), developed mild COVID-19 symptoms that persist for two days only. Although the Pfizer-BioNTech COVID-19 vaccine provoked an immune response that attenuated the severity of the disease among those elderly patients from severe symptoms in non-vaccinated patients to mild symptoms in this vaccinated patient, there was a reduction of the vaccine efficacy against this Brazilian variant [245]. In a study that included 9433 participants who had two doses of ChAdOx1 nCoV-19/AZD1222 vaccine (University of Oxford, AstraZeneca, and the Serum Institute of India), the efficacy of the vaccine on the B.1.1.28 lineage was 73%, and this vaccine had 95% protection rate from COVID-19 hospitalization [246].

### 8.4. B.1.617.2 (Delta Variant)

The B.1.617.2 (delta) variant was first detected in India in December 2020 and became the most reported variant in the country starting in April 2021 then rapidly spread and dominated across the world. It has 10 mutations in the spike protein [247]. In a study that enrolled 156 participants who received one or two doses of BNT162b2 (Pfizer-BioNTech COVID-19 vaccine) or AZD1222 (University of Oxford, AstraZeneca) vaccines. They found that the B.1.617.2 (delta) variant had a reduction in the neutralization titer by 11.3-fold after two doses of Pfizer-BioNTech vaccine, while the reduction was 7.77 and 9.56-fold in B.1.617.1 (Kappa) and B.1.351 (beta) variant respectively. The reduction after two doses of AstraZeneca was 4.01-fold in beta, 1.48-fold in beta, and 0.69 in Kappa variant [248]. Lopez Bernal et al. reported that the efficacy of BNT162b2 (Pfizer-BioNTech COVID-19 vaccine) on delta variant was 35.6% for one dose and 88.0% for the two doses. At the same time, the efficacy of AZD1222 (University of Oxford, AstraZeneca, Oxford, UK) was 30.0% for one dose and 67.0% for the two doses [249]. Although two doses of Pfizer-BioNTech had provoked significantly higher antibody titers than two doses of AstraZeneca, both vaccines had a highly effective level for delta variant after two doses [248,249]. Thus, we recommend two doses of vaccines to provoke the maximum immunity of the vaccine, and there may be a need for a booster dose of vaccines over time to protect from the coming variants.

### 8.5. B.1.1.529 (Omicron)

This variant was first identified and confirmed in Botswana, South Africa, on 9 November 2021. Then it was reported to the WHO on 24 November 2021 [250]. Tulio de Oliveira and his team, who identified this variant, reported that these new mutations in the COVID-19 virus were originated in HIV patients who had the virus for weeks or months [251]. Omicron had 32 mutations in spike protein which help it to be more infectious, at faster rates than other variants’ waves, and may be able to escape from vaccines antibodies [250,251,252]. It is estimated to be 10-fold more infectious than the original virus [253]. Now, it has reached many countries and is still spread rapidly. Accordingly, the WHO and many countries had protective guidelines to limit the spread of this variant by travel restriction toward and from South Africa, or obligatory quarantine for new arrivals, and testing PCR before and after their travels. 

There are no specific symptoms associated with infection by the Omicron variant [254]. Notwithstanding, the Omicron variant has a higher prevalence of asymptomatic patients with high nasopharyngeal viral RNA in comparison to other variants. This may be the major contributory factor for the high and rapid spreading of Omicron worldwide among all populations, even those with a prior COVID-19 infection [255]. However, there was a decrease in severe symptoms or hospitalization rate caused by Omicron variants [256,257]. Still, the rapid spread of this variant could contribute to a cumulative high number of admissions to the hospitals. Omicron may have the ability to evade the available vaccines and has demonstrated reduced vaccine efficacy by twofold more than the Delta variant [253]. However, despite that reduction of vaccine efficacy, the currently available vaccines can protect the population from severe Omicron infection [258]. 

The infection with the Omicron variant can boost and enhance more immune responses than other variants [254]. This means that having a previous natural infection or having the booster dose of vaccines was associated with partial protection against severe Omicron variant symptoms and hospital admissions [257,259,260,261]. A previous COVID-19 infection and having two doses of vaccines can decrease the risk of hospital admission by 50% and 41%, respectively [261]. Having two doses of BNT162b2 (Pfizer-BioNTech COVID-19 vaccine) can provide 33% protection against Omicron variant infection while 80% against Delta variant infection. Additionally, it can decrease severe COVID-19 symptoms that require hospitalization by 70% and 93% against Omicron and Delta variants, respectively [256]. Moreover, the immunity gained by previous COVID-19 infection was found to be less effective against the Omicron variant compared with Delta and Beta variants [256]. This means that Omicron has more ability to reinfect people who have previous COVID-19 infections.

Some subvariants resulted from Omicron and started to spread in a limited way compared to Omicron. Deltacron, BA.2, and IHU variants, which were recently identified in Cyprus, Denmark, and France, respectively, were considered as subvariants from Omicron with not enough available scientific data about them. However, the WHO only considered the omicron variant as a variant of concern (VOC). The early preliminary data predicts that the available vaccines with boosting dose may protect the patients of these subvariants from severe COVID-19 symptoms and hospitalization.

## 9. Is the Booster Dose Useful or Not?

Omicron showed a significant reduction in vaccines-induced neutralizing antibodies for most of the available vaccines (e.g., BNT162b2 (Pfizer-BioNTech COVID-19 vaccine), mRNA-1273 (Moderna vaccine), Ad26.COV2.S (Janssen/Johnson & Johnson vaccine), Sputnik, and Sinopharm) [261]. The effectiveness of two doses of AZD1222 (the Oxford University/AstraZeneca) reduced from 44% against the Delta variant to 5% against the Omicron variant. Similarly, the effectiveness of two doses of BNT162b2 (Pfizer-BioNTech vaccine) reduces from 70% to 19% against Delta and Omicron variants, respectively [261]. However, after giving the booster dose of one of the mRNA-based vaccines (Pfizer/BioNTech or Moderna), the effectiveness raised from 5% to 73% in people who had previously two doses of AZD1222 (the Oxford University/AstraZeneca) and raised from 19% to 77% in people who had previously two doses of BNT162b2 (Pfizer-BioNTech vaccine) [261]. Likewise, Hoffmann M. et al. found that although the Omicron variant has 12 to 44-fold more resistant to BNT162b2 (Pfizer-BioNTech COVID-19 vaccine) than the Delta variant, having either a booster dose of BNT162b2 (Pfizer-BioNTech COVID-19 vaccine) or having two heterologous doses from BNT162b2 (Pfizer-BioNTech COVID-19 vaccine) and ChAdOx1 (Astra Zeneca-Oxford) were found to be more efficient in producing noticeable neutralizing antibodies that can protect against the Omicron variant [262].

Accorsi EK et al. conducted a large case-control study to evaluate the effectiveness of mRNA-based vaccines (Pfizer/BioNTech or Moderna) on Omicron and Delta variants. They divided their samples’ vaccination status into those who were vaccinated with third dose (booster dose) after at least six months from the second dose, those who were vaccinated with only two doses before at least six months (who didn’t have the booster dose although they were eligible for it), and those who were not vaccinated. They included 23,391 American adults as a case group (56.0% of them have been infected with Omicron variant and the rest with Delta variant) and 46,764 as a control group. They found that receiving three doses of mRNA-based vaccines (either Pfizer/BioNTech or Moderna) was associated with a higher protection rate against Delta and Omicron variants compared with receiving only two doses of vaccines or being unvaccinated. Pfizer/BioNTech and Moderna vaccines showed more protection rate against the Delta variant compared with the Omicron variant [263]. 

Garcia-Beltran WF et al. found that most vaccines (which was Pfizer/BioNTech, Moderna, or Johnson & Johnson in their study) were unable to produce detectable neutralization against Omicron variant unless boosted with either one of mRNA-based vaccines (Pfizer/BioNTech or Moderna). They found that three doses of any mRNA-based vaccines (Pfizer/BioNTech or Moderna) were significantly associated with good neutralization against Omicron and Delta variants. Moreover, adenovirus vectored vaccine (Ad26.COV2.S vaccines which is Janssen/Johnson & Johnson vaccine) was significantly associated with higher neutralization against Omicron and Delta variants if boosted with mRNA-1273 (Moderna vaccine) rather than boosted with Ad26.COV2.S vaccine alone [264].

Costa Clemens SA et al. conducted their study on 1240 Brazilian adults who had two doses of Sinovac vaccine before six months. They divided the participants into four groups where they received a third (booster) dose, either homologous dose of Sinovac vaccine or heterologous dose from BNT162b2 (Pfizer-BioNTech COVID-19 vaccine, which is mRNA based-vaccine), Ad26.COV2.S vaccine (aka Janssen/Johnson & Johnson vaccine, which is adenovirus vectored vaccine), or AZD1222 vaccine (aka Astra Zeneca-Oxford, which is recombinant adenoviral-vectored ChAdOx1 nCoV-19 vaccine). They found that the neutralizing antibodies before taking any booster dose were 20.4% and 8.9% among participants aged 18–60 years and older than 60 years, respectively, after six months of taking two doses of Sinovac vaccine. However, there was a significant increase in neutralizing antibodies against Delta and Omicron variants after 28 days from taking the booster dose for all types. 

Moreover, a heterologous booster dose can enhance more potent protection and neutralizing antibodies compared with a homologous booster dose [265]. This agrees with the results of Pérez-Then E, et al., who found that having a heterologous booster dose of BNT162b2 (Pfizer-BioNTech COVID-19 vaccine) for participants who had primary two doses of Sinovac vaccine, can cause increased neutralizing antibodies against Delta variant to be similar to those who received two doses of BNT162b2 vaccines and can increase the antibodies by 1.4-fold against Omicron variant compared to two doses of BNT162b2 vaccines [266].

In the COV-BOOST study, which was a large, blinded, multicenter randomized, control trial conducted in the United Kingdom, seven vaccines were given as a third booster dose for 2878 adult participants aged more than 30 years and primarily received two doses of BNT162b2 (Pfizer-BioNTech COVID-19 vaccine) or ChAdOx1 nCoV-19 (Astra Zeneca-Oxford vaccine). They used seven booster dose vaccines, one of the following: BNT162b2 (Pfizer-BioNTech), a half dose of BNT162b2 (Pfizer-BioNTech), mRNA-1273 (Moderna vaccine), Ad26.COV2.S vaccine (Janssen/Johnson & Johnson vaccine), Novavax, a half dose of Novavax, or ChAdOx1 nCoV-19 (Astra Zeneca-Oxford vaccine). Although there were some differences in neutralizing antibodies produced by the booster dose, all vaccines showed good immunological response with no threat to the participants’ safety. Moreover, the best immunogenicity was with a booster dose of Moderna, then Pfizer-BioNTech, then half dose of Pfizer-BioNTech, then the rest of vaccines for both who primary received two doses of Astra Zeneca-Oxford or Pfizer-BioNTech vaccines. The highest immune response was among participants who received two primary doses of Pfizer-BioNTech followed by Moderna booster dose [267].

On the other hand, Singhal T stated that giving booster doses of vaccines can disrupt the global equity in vaccines distributions among the countries with no marked impact on controlling the rapid spread of the Omicron variant [260].

## 10. Conclusions

COVID-19 became the most significant battle in health care in recent history and required sufficient global coordination and response to address. Vast efforts were conducted to produce efficient vaccines and treatments. Consequently, there is progress in the reduction of morbidity and mortality rates. However, the continuous appearance of SARS-CoV2 variants is a major challenge worldwide. Therefore, further efforts are recommended to be taken. All eligible people have at least the full doses of the available vaccine in their country. The WHO and the global committees should make more efforts to distribute vaccines for all countries in the world, especially low-income countries. This can lead to controlling and decreasing the number of COVID-19 patients, severity of infection, and mortality, in addition to decreasing the hot spots country from generating new resistant variants. Moreover, we recommend using heterologous vaccines—if available— with different mechanisms of action. 

We strongly encourage taking a booster dose every six months from the date of the last vaccine with a preferable choice of using a heterologous dose, especially one of the mRNA-based vaccines (Pfizer/BioNTech or Moderna). This is because using a strategy based on a heterologous vaccine can increase immunogenicity and neutralize antibodies against mutated SARS-CoV-2 variants. More efforts are required for continuous testing and evaluating the effectiveness of the currently available vaccines on any new SARS-CoV-2 variant. Consequently, the international authorities should urgently report any emergent or suspected mutation to the WHO. In addition to the rapid investigations, research, and data collection and make them available for the public, investigators, and researchers. Finally, hand hygiene, well-fitting masks, physical distancing, avoiding full and crowded places are the simple requirements for everyone to minimize the risk of infection of any variants and its complications.

## Figures and Tables

**Table 1:** Status of COVID-19 Vaccines until 30 November 2021 [184,186,187,188,189,190,191,192,193,194,195,196].

	Manufacturer	Name of Vaccine	Platform	Dossier Accepted for Review *	Status of Assessment **	Predicted Decision Date ***
1	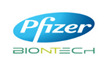	BNT162b2/COMIRNATY Tozinameran (INN)	Nucleoside modified mRNA.	√	Finalized	31 December 2020
2	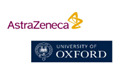	AZD1222	Recombinant ChAdOx1 adenoviral vector encoding the Spike protein antigen of the CoV.	Accepted core data of AZ—non-COVAX.	Core data—now as a donation for COVAX.	15 April 2021
				Data for Covax is expected in April 2021.	Awaited	April 2021
3	SKBIO 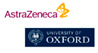	AZD1222	Recombinant ChAdOx1 adenoviral vector encoding the Spike protein antigen of the CoV.	√	Finalized	15 February 2021
4	Serum Institute of India	Covishield (ChAdOx1_nCoV19)	Recombinant ChAdOx1 adenoviral vector encoding the Spike protein antigen of the SARS-CoV-2.	√	Finalized	15 February 2021
5	Sinopharm/BIBP1 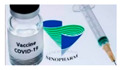	SARS-CoV-2 Vaccine (Vero Cell), Inactivated (lnCoV)	Inactivated, produced in Vero cells.	√	In progress	Finish in April 2021
6	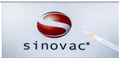	SARS-CoV-2 Vaccine (Vero Cell), Inactivated	Inactivated, produced in Vero cells.	√	In progress	Early May 2021
7	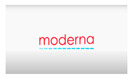	mRNA-1273	mRNA-based vaccine encapsulated in a lipid nanoparticle (LNP).	√	In progress	End April 2021
8	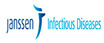	Ad26.COV2.S	Recombinant, replication-incompetent adenovirus type 26 (Ad26) vectored vaccine encoding the (SARS-CoV-2) Spike (S) protein.	√	Finalized	12 March 2021
				√	Awaited	For fixing after submission of data
9	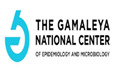	Sputnik V	Human Adenovirus Vector-based COVID-19 vaccine.	“Rolling” submission of clinical and CMC data has started.	Additional data is required.	Will be fixed after all data is submitted and completed.
10	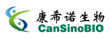	Convidecia	Ad5-nCoV	Rolling data starting April 2021	Finalized	7 September 2021
11	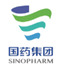	Sinopharm/WIBP	Inactivated SARS-CoV-2 Vaccine (Vero Cell)	√	Finalized	25 February 2021
12	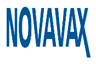	Novavax	Subunit COVID-19 vaccine	√	Finalized	4 August 2021
13	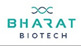	Covaxin	Inactivated virus-based COVID-19 vaccine	√	Finalized	3 November 2021
14	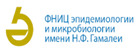	Sputnik light	Ade26 vector	√	Finalized	6 April 2021
15	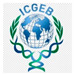	Abdala	Protein subunit	√	Finalized	9 July 2021
16	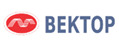	EpiVacCorona	Peptide-based vaccine	Rolling data starting December 2020	Not yet	
17	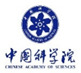	Zifivax	Adjuvanted protein subunit COVID-19 vaccine	√	Finalized	7 September 2021
18	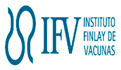	Soberana 02	conjugate	√	Not yet	
19	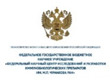	CoviVac	Inactivated virus-based COVID-1 9Vaccine	√	Finalized	20 February 2021
20	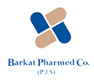	COVIran	Inactivated virus-based COVID-1 9Vaccine	√	Finalized	13 June 2021
21	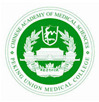	Chinese Academy of medical sciences COVID-19 vaccine (IMBCAMS COVID-19 VACCINE)	Inactivated virus-based COVID-1 9Vaccine	√	Finalized	9 June 2021
22	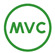	MVC COVID-19 vaccine	Protein-subunit COVID-19 vaccine	√	Not yet	
23	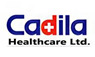	ZyCoV-d	DNA plasmid-based Covid-19 vaccine	√	Finalized	1 July 2021
24	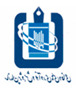	FAKHRAVAC	Inactivated virus-based COVID-1 9Vaccine	√	Finalized	9 September 2021
25	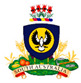	COVAX-19	Recombinant-protein based COVID-19 vaccine	√	Finalized	6 November 2021

* Dossier Submission dates: more than one date is possible because of the rolling submission. The dossier is accepted for submission after the screening of the received submission. ** Status of assessment: 1. Under screening; 2. Under assessment; 3. Waiting for responses from the applicant. 4. Risk-benefit decision 5. Final decision made. *** Predicted decision date: this is only an estimate because it depends on when all the data is submitted under rolling submission and when all the responses to the assessors’ questions are submitted.

## Data Availability

Not applicable.
